# Which online format is most effective for assisting Baby Boomers to complete advance directives? A randomised controlled trial of email prompting versus online education module

**DOI:** 10.1186/s12904-017-0225-9

**Published:** 2017-08-29

**Authors:** Sandra L. Bradley, Jennifer J. Tieman, Richard J. Woodman, Paddy A. Phillips

**Affiliations:** 1 School of Nursing and Midwifery, Flinders University South Australia, GPO Box 2100, Adelaide, SA 5000 Australia; 2 School of Health Sciences, Flinders University, South Australia, GPO Box 2100, Adelaide, SA 5000 Australia; 3 Epidemiology and Biostatistics, School of Medicine, Flinders University South Australia, GPO Box 2100, Adelaide, SA 5000 Australia; 4 School of Medicine, Flinders University South Australia, GPO Box 2100, Adelaide, SA 5000 Australia

**Keywords:** Baby Boomers, Advance care directives, Email, Online, Education, Randomized controlled trial, Completion rates

## Abstract

**Background:**

Completion of Advance Directives (ADs), being financial and healthcare proxy or instructional documents, is relatively uncommon in Australia. Efforts to increase completion rates include online education and prompting which past literature suggests may be effective. The aim of this randomized controlled trial was to assess computer-based online AD information and email prompting for facilitating completion of ADs by Australian Baby Boomers (b.1946–1965) as well as factors which may impede or assist completion of these documents by this generation when using the online environment.

**Methods:**

Two hundred eighty-two men and women aged 49–68 years at the time of the trial were randomly assigned to one of 3 intervention groups: education module only; email prompt only; email prompt and education module; and a control group with no education module and no email prompt. The randomized controlled trial was undertaken in participants’ location of choice. Randomization and allocation to trial group were carried out by a central computer system. The primary analysis was based on a final total of 189 participants who completed the trial (*n* = 52 education module only; *n* = 44 email prompt only; *n* = 46 email prompt and education module; and *n* = 47 control). The primary outcome was the number of individuals in any group completing any of the 4 legal ADs in South Australia within 12 months or less from entry into the trial. Frequency analysis was conducted on secondary outcomes such as reasons for non-completion.

**Results:**

Mean follow-up post-intervention at 12 months showed that 7% of overall participants completed one or more of the 4 legal ADs but without significant difference between groups (delta = 1%, *p* = .48 Prompt/Non-Prompt groups, delta = 5%, *p* = .44 education/non-education groups). Reasons offered for non-completion were too busy (26%) and/or it wasn’t the right time (21%).

**Conclusion:**

Our results suggest that neither email prompting nor provision of additional educational material online were sufficient to significantly impact AD completion rates for this generational cohort. Research with this cohort over longer periods of time exploring online preferences for engagement with ADs as they age may provide better insight into using this environment for ADs with this group.

**Trial Registration:**

Australian New Zealand Clinical Trials Registry ACTRN12616000425493.

**Electronic supplementary material:**

The online version of this article (doi:10.1186/s12904-017-0225-9) contains supplementary material, which is available to authorized users.

## Background

With the intent of supporting the autonomous future healthcare decision-making of the person [1], many researchers and policy makers around the world have tried various methods to increase the uptake and completion of healthcare advance directives (ADs) [2–4]. These documents provide proxy or instructional decision-making for a future catastrophic medical event when the person who has completed the directive is unable to relate their preferred healthcare treatment [4, 5].

In Australia, there has been a national push to increase the completion of these documents through information campaigns such as Planning Ahead Day and Dying to Know Day [6, 7]. These campaigns, and others like them, use a range of formats, including online methods, to encourage the public to formalise future healthcare arrangements. Online methods for disseminating and promoting healthcare ADs include the use of Massive Open Online Courses (MOOCs) [8]; video decision aids [9]; computer-based interactive means of completing ADs [2, 9]; and applications (Apps) for information and education on healthcare ADs and its corollary, advance care planning (ACP) [10]. These online formats, although an increasingly important method of engagement in this area, have not yet been shown unequivocally to lead to increasing completion rates of healthcare ADs [2, 9, 11, 12]. This has led to lingering debate about the effectiveness of healthcare ADs to direct future care in the manner intended or expected [3, 4, 11–13].

Measuring whether online formats or other promotional efforts to increase healthcare AD completions have been successful may be determined through completion rates but this measure is not used consistently [14]. Examples of research exploring the effectiveness of different online methods for increasing completion rates of healthcare ADs have included: email prompting of physicians to discuss healthcare ADs with specific patient types [15]; computer-based, interactive online formats which allow for online completion of healthcare ADs [16] and online decision aids for specific patient and non-patient groups leading to completed healthcare ADs or advance care plans [17]. Research evaluating the effectiveness of these online interventions has shown that they may be helpful in specific contexts and specific outcomes but may not translate to a broader and more general audience [17]. Klugman and Usatine [16] discovered that when online websites are used to promote healthcare AD completions for a broader audience, these formats run the risk of premature collapse when funding initiatives expire and defeat efforts to enhance completion rates or engagement with ADs.

Factors to consider when choosing online formats to engage a broader audience for promotion of healthcare AD completions include age and comfort in using the online environment. Recent research by the Australian Bureau of Statistics [18] showed that Internet use was lowest amongst those 65 and older (51% compared to 99% of those aged 15–17) while research by Bradley et al. [19] and White et al. [20] showed that AD completions in Australia begin to increase from the age of 50 and above. So, how successful are online formats for increasing AD completion rates in a population group as it ages but who may use the online environment less routinely?

Research by several Australian government agencies [21, 22] showed that the Baby Boomer generation (b. 1946–1965 in Australia and aged 47–67 at time of study [23]) were increasingly using the Internet for email and research/news/general browsing with both activities providing a conduit to receive information about ADs as well as access to online forms. Knowing this, we decided to conduct a randomized controlled trial (RCT) on this generational group to see which of two online formats would facilitate an increase in AD completions. The aim of our RCT was to test email prompting or online education for facilitating AD completions in this Baby Boomer group as well as expose any factors that impeded or assisted them in this process.

## Methods

### Study design and oversight

Combining an understanding of advance directive use, population demographics and online formats, this article reports on the results of a randomised controlled trial conducted in Australia. The aim of the trial was to measure the number of Baby Boomers completing any type of AD when exposed to one of two online mechanisms, an email prompt or a computer-based, interactive online format. The number of individuals in any of the intervention groups completing any AD was compared to individuals who were not exposed to these online interventions. Factors impeding or assisting completion of these documents are reported as a secondary outcome of the trial.

A 12-month, 2 × 2 factorial RCT design was used to test the effectiveness of email prompting, online education or both for improving the number of individuals completing any legal AD in South Australia. Both financial and healthcare ADs were measured as previous research by Bradley et al. [24], Bradley [25] and White et al. [20] indicated a link between financial and healthcare AD completions. The RCT was conducted between April 2013 and June 2014. The null hypothesis for this RCT was that exposure to any intervention would not significantly increase the number of individuals completing any one of 4 legal South Australian Advance Directives (Enduring Power of Attorney, Enduring Power of Guardianship, Medical Power of Attorney or Anticipatory Direction) when compared to those not exposed to an intervention.

All authors participated in the design of the study and writing of the manuscript.

### Ethics

All participants were provided with an Information Sheet about the study and provided informed consent prior to participation. All data collection was conducted through a third-party data management system (Research Data Management System of CareSearch) ensuring privacy and confidentiality for participants in the processing of results. The researcher was blinded to participant identification at the time of group allocation via a unique identification number without identifying personal details. At the conclusion of the trial, to provide equity and fairness, all participants, regardless of group allocation, received all information contained within the trial about South Australian ADs.

The study was approved by the Flinders University Social and Behavioural Research Ethics Committee (Project No. 6069) and has been registered with the Australian and New Zealand Clinical Trial Registry (ACTRN12616000425493).

### Setting

Participants used their own computer and Internet resources at a location of their choice. Distribution of materials was conducted through the online environment via participant’s nominated email address. No face-to-face or on-site setting was used with all correspondence conducted through email.

### Participants

Participants were recruited for the trial via South Australian newspaper and magazine advertisements, organisational and university email newsletters, hard copy fliers posted in libraries and community centres and radio advertisement. Participants were included in the trial if they met the following criteria: born 1946–1965; South Australian resident; had not completed any of 4 legal proxy/instructional ADs (Enduring Power of Attorney, Enduring Power of Guardianship, Medical Power of Attorney or Anticipatory Direction); had computer and Internet access and a valid email address; were fluent in written English; and, were able to participate in the study over the course of 12 months.

### Randomisation and interventions

Participants were randomised into intervention or non-intervention groups using stratified block randomisation in order to minimise differences between gender, location (metropolitan/rural) and age (1st or 2nd decade of Baby Boomers: 1st decade = 1946–1955; 2nd decade = 1956–1965). The block randomisation ensured equal balance in subject numbers across the four groups: control (no email prompt, no online education module – Group A); online education module only (Group B); email prompt only (Group C); both email prompt and online education module (Group D) (Additional file 1: Figure S1).

At the beginning of the trial, all participants received a pre-survey containing 21 questions on legal and non-legal AD use, substitute decision-making, online engagement, and demographic information (Additional file 2: Figure S2). Upon receipt of the completed pre-survey, the participant was then allocated to a group.

Groups B and D received information about South Australian ADs via a specially-designed computer-based, interactive online education module for the project a sample page of which is shown in (Additional file 3: Figure S3). Access to the module was provided via a link embedded within an email to participants in these two groups only at the beginning of the trial with the link accessible throughout the trial. The education module included information about all 4 South Australian ADs, why a person might wish to consider writing an AD as well as additional information about changes to be enacted in South Australia in relation to a new version of advance care directive. This online education module was housed within CareSearch and was not made available to the general public.

Groups C and D received the email prompt intervention which consisted of 3 email surveys asking if participants had completed an AD. The email prompt design arose from suggestions by participants in a Masters thesis [25] by the first author which indicated that prompting would be a way of bringing to front of mind completion of the document. The email surveys were sent at various time points throughout the study depending on when the participant was recruited with the 3rd email survey sent to all participants in groups C and D at the same time (2 months before the end of the study) as full recruitment had been reached by that stage. These email surveys contained the same questions each time with a different prompt message about ADs contained within the introduction to each email survey (Additional file 4: Figure S4).

The control group (A) received only the pre-survey. No additional information or prompting was provided throughout the duration of the trial.

Twelve months after the trial began and interventions had ceased, a post-survey (Additional file 5: Figure S5) was sent to all remaining participants in the trial across all 4 groups. The post-survey included questions similar to the pre-survey.

### Study outcomes

The primary outcome for this study was the number of individuals within each group self-reporting completion of any of the 4 legal SA ADs from a baseline of 0. Secondary outcomes explored reasons for or against completion of ADs; comfort with and use of computers and other electronic devices; preferences for online formats for information on ADs; whether participants assisted others with ADs, as well as those who assisted participants with AD information during the trial.

### Sample sizes

Sample sizes for the trial were calculated on a baseline completion rate in the control group of 1% and a completion rate in any of the intervention groups of 10% with 80% power and a two-sided type 1 error rate of alpha = .05. For a parallel group design this would require *n* = 100 subjects per group (*n* = 400 total) but the 2 × 2 design with testing of the 2 main effects allows this to be reduced to *n* = 50 per group for a total sample size of *n* = 200. We allowed for approximately 20% attrition rate. Recruitment was then based on *n* = 282 participants (68 participants per group).

### Data analysis

Surveys with the same UID number were used for comparison of individual pre- and post-survey results. Comparison of the 4 groups for AD completion rates was performed using chi-squared test of association. Effectiveness of the different intervention groups to increase individuals completing ADs was assessed using binary logistic regression and the primary outcome of AD completion rates is reported as a percentage and 95% confidence interval. Treatment effects for the two interventions were assessed using main effects, i.e. with 2 binary indicators, one for each of the 2 separate treatments. An interaction term between the 2 treatments was also assessed in separate models to test whether the effects of one treatment depended upon receiving the other. This yielded Prompt/Non-Prompt groups and education module/Non-education module groups for representation of the results. All other analyses were conducted on a per protocol basis with a two-side probability value of .05 constituting a statistical significance. Statistical analyses were conducted using SPSS version 19. Frequency analysis only was used for analysing secondary outcomes.

## Results

The aim of this randomized controlled trial was to assess whether computer-based online AD information and email prompting could facilitate completion of ADs by Australian Baby Boomers (b.1946–1965). Factors impeding or assisting completion were also assessed as secondary outcomes.

Of the 282 participants randomised into the 4 groups throughout the trial, 93 withdrew (33%) leaving 189 participants for data analysis as seen in the CONSORT diagram in (Additional file 6: Figure S6). Reasons for dropping out included health issues, time constraints, no longer interested, and having already completed one of the 4 legal ADs after registration to participate but before pre-survey commenced. The 4 groups of participants are described individually as well as in configurations of Prompt (Groups C + D)/Non-Prompt (Groups A + B); education module (Groups B + D)/Non-education module (Groups A + C) with results represented in (Additional file 7: Table S1 and Additional file 8: Table S2 respectively).

The demographic profile of the 189 participants who completed the trial and both pre- and post-surveys is described in Table S1. The proportion of females participating was 75% compared to 25% males and the majority of participants were from the metropolitan area (76%) and were married (61%). From the total number of initial participants (*n* = 282), the final rate of both pre- and post-survey completion across all groups was 67% (*n* = 189/282). On a per group basis, pre- and post-survey completion was: Group A, 65% (*n* = 47/72); Group B, 72% (*n* = 57/72); Group C, 61% (*n* = 44/72); and Group D, 64% (*n* = 46/72).

### Computer based online education vs email prompting and AD completion rates

Of the final number of participants who completed both the pre- and post-survey, Table S2 shows that 7% (*n* = 13/189) completed at least one of the 4 legal South Australian ADs over the course of the trial. Documents completed most often across all groups were the financial document (Enduring Power of Attorney (EPA), *n* = 7) and the proxy healthcare documents (Enduring Power of Guardianship (EPG), *n* = 6; Medical Power of Attorney (MPA), *n* = 5). Instructional healthcare documents were completed rarely (*n* = 2 for Anticipatory Direction and *n* = 2 for Living Will).

Comparison of completion rates between the combined groups was performed using both chi-squared test of association and binary logistic regression. Table 1 shows that there was no significant difference in the AD completion rates for email prompt (Group C + D) vs no prompt (Group A + B) respectively for any AD document (6%, *n* = 16 vs 7%, *n* = 13, *p* = .48).

**Table 1:** Completion of any AD Documents (*N* = 189)

Q2A: Completion of any of 4 Individual AD documents							
	Total of Individuals who completed any AD document (*N* = 189) n^ab^ (%)	Prompt Group (C + D, *N* = 90) n^ab^ (%)	No-Prompt Group (A + B, *N* = 99) n^ab^ (%)	*P* Value	AD Module Group (B + D, *N* = 98) n^ab^ (%)	No-AD Module Group (A + C, *N* = 91) n^ab^ (%)	*P* Value
Any AD Document (+)	13 (7%)	6 (6%)	7 (7%)		9 (9%)	4 (4%)	
No AD Document (−)	176 (93%)	84 (94%)	92 (93%)	.48	89 (91%)	87 (96%)	.44

^a^number who completed or didn’t complete

^b^N = rounded to whole number

Similarly, there was no significant difference in the AD completion rates for education module (Group B + D) vs no-education module (Group A + C) for any AD document (9%, *n* = 13 vs 4%, *n* = 16, *p* = .44). Binary logistic regression showed no statistically significant difference in completion rates for any of the 4 documents between combined groups (Additional file 9: Table S3). Specifically, there was no significant difference in the AD completion rates for email prompt vs no prompt for EPA (2% vs 2%, *p* = .82); EPG (1% vs 2%, *p* = .49), MPA (1% vs2%, *p* = .24) or Anticipatory Direction (1% vs 2%, *p* = .95). Similarly, there was no statistically significant difference in the AD completion rates for education module vs no education module for EPA (3% vs 1%, *p* = .11), EPG (2% vs 1%, *p* = .47), MPA (1% vs 2%, *p* = .58) or Anticipatory Direction (1% vs 1%, *p* = .94).

### Secondary outcomes

#### Factors associated with AD completions

Participants recorded completing other documents associated or thought of as ADs during the trial. These documents included Wills (72%) and Organ Donation Cards (49%).

When completing documents, participants indicated that they were assisted most often by a lawyer (42%) or family members (21%). Healthcare professionals or spiritual guides were rarely used for assistance (1% or less overall). However, (Additional file 10: Figure S7) shows that when discussing ADs with others, participants indicated that they were more likely to speak with family (88%), friends (42%) and work colleagues (15%) than lawyers (6%), doctors (5%) or financial planners (3%).

Only 10% of the participants assisted someone else with an AD during the course of the trial. The EPA (9%), EPG (10%) and Will (10%) were the documents participants most often assisted with. Less than 5% of participants assisted with any instructional healthcare AD (*n* = 9 for Anticipatory Direction and Living Will; *n* = 5, advance care plan). Only 16% of participants recorded acting as a substitute decision-maker during the trial period with most acting as a substitute decision-maker under the EPA (12%) or MPA (4%).

#### Factors Impeding or assisting with AD completion

In total, 47% of participants indicated they were highly likely or in the process of completing an AD while 1% responded that they were not interested in completing an AD at all (Additional file 11: Figure S8). No participant indicated that ADs were against their religious or cultural beliefs.

Additional file 11: Figure S8 shows the most common reasons chosen for not completing an AD during the trial with *too busy* (26%) and *not the right time* (21%) being the most common. Other factors influencing completion were: *not having anyone to discuss ADs with* (10%); *needing more information* (10%); *didn’t feel the need to complete* (10%); *unable to access the AD form directly* (4%); and *not being able to choose a substitute decision-maker* (4%).

A majority of participants (53%) indicated they would act as a substitute decision-maker for someone else while 37% indicated it would depend on who asked. Only 1% indicated they would not act as a substitute decision-maker.

### Influence of the online environment for AD completions

#### Online comfort and use

The pre-survey responses indicated that 45% of all participants had a 100% comfort level with computer use with the remainder expressing comfort levels at 80–90% (Additional file 12: Figure S9). Additional questioning in this area showed that most participants used a desktop computer (74%), laptop (67%) and a smartphone (59%). When seeking information about ADs, 42% of participants used their desktop computer with 25% using a laptop, 10% a smartphone and 34% not using any online device at all.

Post-survey responses indicated that 41% of participants found information about ADs on the Internet helpful during the trial but only 28% indicated the Internet was helpful for actual completion of ADs. Forty-two per cent of participants indicated a preference for online AD forms after the trial.

#### Email prompting

Additional file 13: Figure S10 illustrates when participants would like to be prompted to complete an AD. A majority of participants indicated a preference to be prompted by email to complete ADs on their birthday (43%) with a preference for 60th (13%) or 70th (14%) birthdays. Other preferences included prompting in affiliation with Will completion (36%), when seeing a financial planner (21%) or completing an Organ Donation Card (21%). Email reminders were more favoured (74%) than SMS text message (21%), Facebook (15%) or television reminders (13%) while 12% of participants did not want to be prompted at all.

#### Education module

Additional file 14: Figure S11 shows that 49% of participants did not feel that the education module encouraged them to complete ADs. It did not appear that this response was affected by terminology or content with 58% of participants responding that they would recommend the education module to family or friends and 22% indicating that the information within the education module met their needs. In addition, 54% of participants indicted they now knew more about ADs as a result of participating in the trial however more nuanced responses indicated that: 42% of respondents wanted direct access to forms; 30% wanted more information on when to complete them; 26% wanted guidelines for choosing a substitute decision-maker; 18% wanted testimonials and instructions on how to have conversations about ADs; and 17% wanted better reasons or explanations as to why ADs should be completed.

## Discussion

The argument has been made that the online environment can assist with AD creation by providing a readily accessible format for those who are comfortable with computer use and the online environment [2, 9]. The aim of our RCT was to test this hypothesis by comparing the effectiveness of two online interventions, an online education module and email prompting, to facilitate AD completion in a generational group which has become accustomed to the online environment. The specific purpose for conducting this RCT in this manner was to test a similar view expressed by participants in an earlier study by Bradley [25] in which Baby Boomers in South Australia indicated that they would be more willing to complete ADs if they had: experience and knowledge of the forms; could name a substitute decision-maker; and could have easy access to information and the forms at a time of need as well as being prompted to complete them. Our RCT tested the latter premise; that is, facilitation of completion of ADs by making information available in an easy to access format (the online environment) at a time of the person’s preference (after an email prompt). Our RCT also assessed factors that impeded or assisted this generational group to complete ADs using these online formats.

Results of our study indicate that the Baby Boomer participants were comfortable with using the online environment and being prompted to complete ADs. However, access to readily accessible information and reminders were not enough to facilitate completion to the 10% level of effect anticipated. A limitation of this study is that participants could not access directly online AD forms; this was nominated as their preferred mechanism for AD completion. Because this was not possible at the time this study was conducted, our results may be lower than would be the case if the online forms had been available. Research is now being conducted by the South Australian Department of Health to ascertain how effective the free, online form that has been made available since 2014 [26] is for increasing completion rates and will indicate whether direct access to this online document has been enough to increase completion rates of healthcare ADs in South Australia above the 10% previously described [19].

Halpern [11] and Lewis et al. [12] have argued that until higher levels of evidence are obtained in research studies of the effectiveness for interventions to actually increase healthcare AD completions to a significant level, there remains doubt as to whether these documents can assist patients, families and healthcare professionals with future healthcare treatment decisions. The strength of this study is that our RCT contributes to the higher level of evidence sought; however, our study, like many of those before it, has not been able to replicate previous RCTs due to the specificity of information required that is legal and relevant to the community involved. This means that generalisability of our results is lacking although, heterogeneity notwithstanding, our results provide further evidence that online formats alone will not enhance AD completions [9, 27].

Fagerlin & Schneider [13] argue that completion rates only measure the willingness of people to engage with these documents rather than success in translating patient wishes into actual anticipated outcomes; therefore, completion rates should only be viewed as an indicator of willingness to complete rather than effectiveness of the instrument. Our study would seem to support that view. Although a limitation of our study is that we did not have a naïve control group without pre-disposition to complete ADs, nevertheless, the initial enthusiasm for participation in the study (over 681 enquiries) indicated that people had been contemplating the subject long enough to seek an incentive (prompting or information) to further consider completion of ADs. Nevertheless, simply by participating in the study and/or being subject to the pre-survey may have created levels of completion above the number that would have been completed by a naïve audience. Durbin et al. [28] note in their systematic review the lack of this type of control in randomised controlled trials on this subject.

Nevertheless, results from our study provide original evidence for this particular generational cohort of reasons why people may be interested in completing ADs or information around them but still fail to complete them. Reasons provided include those which have previously been identified in the literature as practical, technical and emotional considerations [13] as well as research design [11, 12]. Factors indicated by our participants as preventing them from completing ADs beyond the ability to access the form online were *being too busy* and it not being *the right time*, neither of which is directly affiliated with ease of access or prompting but rather the more emotional considerations proffered by Fagerlin and Schneider [13].

Not being prepared to complete these documents until the right time does not support the efforts for promotion of these documents to be done as early as possible [5]. In particular, participants in our study nominated their 60th or 70th birthdays as being more reasonable times to contemplate these documents which may be too late if a sudden, traumatic medical event arises beforehand.

Lewis et al. [12] and Brinkman – Stoppelenburg et al. [3] identified that the use of ADs to initiate conversations and discussion about future healthcare or end of life care is valuable; however, the evidence is insufficient to suggest that formalised ADs improve healthcare professional engagement or discussions of end of life care at the time of need [29–31]. Participants in this study indicated little engagement with healthcare professionals when considering or completing ADs preferring instead to discuss these documents with family, friends or work colleagues. Although advance care planning emphasises having conversations with healthcare professionals in addition to or instead of dependence on ADs [3, 12, 30], our study showed that this was unlikely to happen in the initial stages of AD consideration by a group of people who may not be in the throes of serious or terminal illness. Research by Tilse et al. [29] and White et al. [20] indicate that for a group like the Baby Boomers who are nearing retirement, estate planning through Wills and Enduring Powers of Attorney creation may be more influential in establishing the first steps towards other AD completions. Our study showed a high propensity for Will completion by this group of participants although it did not lead to anywhere near the same level of completion of healthcare ADs. The reasons for this remain unclear but more targeted research on people with a particular disease [32, 33] or level of illness [34] indicate that confronting mortality itself may inhibit contemplation of creation of ADs until closer to the event. Without documented healthcare AD instructions, decision-making for medical crisis situations may be left to healthcare professionals and others who are not aware of the individual’s preferred care leading to unnecessary, intrusive and costly interventions that were not what the person would have wanted [35, 36].

Encouragement to discuss options earlier rather than later has led to an increased emphasis on ACP [3, 30, 32–34] with additional online formats to prompt these conversations [28]. Websites such as Start2Talk [36] by the Alzheimer’s Association in Australia can assist with conversational elements of ADs but as yet there is insufficient evidence to suggest that the process of ACP through use of an online website results in completed documents that protect a person’s autonomy at the time of need. Indeed, evidence by Blackler [37] shows the influence of family can compromise enactment of ADs while Nabozny et al. [38] have shown that healthcare professionals can limit the choices offered when engaging in AD conversations; both situations serve to undermine informed and autonomous decision-making or the completion of an AD. When the use of facilitation for AD completions has been shown to be effective, it is usually within an environment of advance care planning [39] which the majority of Baby Boomers would not be involved in until the time of need. Therefore, in a wider sense, this trial showed that the Baby Boomer generation may be ready to engage in contemplation of these documents but not yet actual completion. Our study shows that simply providing information or prompts in an online environment will be insufficient to increase AD completion rates in this generational group as they are too busy and it isn’t the right time for them to do so. However, other elements of our research indicate potential for the online environment to be more useful in this regard if associated with estate planning. Online formats which encourage discussion of ADs through ACP will facilitate families and friends to keep this issue ‘front of mind’ for when the time is right.

## Conclusion

Our RCT demonstrated that neither email prompting nor provision of additional educational material online were sufficient to significantly impact AD completion rates in this generational group. Nevertheless, our study is one of few which has explored online formats for facilitating completion of ADs in a specific population demographic without reference to disease or illness status. In this study, we have shown that although the online environment, in and of itself, may not yield an increase in completion of ADs at the point of contact, nevertheless, it provides the opportunity for a generation entering older age to contemplate the need and creation of these documents through discussion with family and friends. As more of these documents enter the healthcare sector, other aspects of AD use which may assist in increasing completion rates, such as examples of how and when to complete them, and increased conversations and discussions with those likely to be involved would benefit those wishing to complete these documents at a preferred time. Results from this trial indicate that Baby Boomers are keen to learn about ADs even if they are not quite ready to complete them, yet.

## Additional files

Additional file 1: Figure S1. Diagram of 2 × 2 factorial design of RCT. (DOCX 12 kb)

Additional file 2: Figure S2. Pre-survey for RCT. (DOCX 22 kb)

Additional file 3: Figure S3. Sample page from online education module. (DOCX 119 kb)

Additional file 4: Figure S4. Email Survey sent as Prompt for completing AD. (DOCX 15 kb)

Additional file 5: Figure S5. Post Survey (DOCX 21 kb)

Additional file 6: Figure S6. Flow diagram of inclusions and exclusions based on CONSORT recommendations. (DOCX 107 kb)

Additional file 7: Table S1. Demographics of 189 Participants in RCT. (DOCX 20 kb)

Additional file 8: Table S2. Primary outcomes of individuals and individual documents completed per group from Pre-survey to Post-survey. (DOCX 14 kb)

Additional file 9: Table S3. Binary logistic regression analysis comparing Prompt/Non-Prompt v AD module/non-AD module groups AD completions (*N* = 189). (DOCX 13 kb)

Additional file 10: Figure S7. Type of Person with whom ADs were Discussed (*N* = 147). (DOCX 1069 kb)

Additional file 11: Fig. S8. Reasons for Not Completing an AD during the RCT (*N* = 189). (DOCX 382 kb)

Additional file 12: Figure S9. Level of comfort individual has with using computer on a scale of 0–100%. (DOCX 2170 kb)

Additional file 13: Figure S10. Best time for a Reminder. (DOCX 1127 kb)

Additional file 14: Figure S11. Document Completions influenced by AD Module (*N* = 189). (DOCX 1056 kb)

## Abbreviations

ACP: Advance care planning
AD: Advance directive
Ant Dir: Anticipatory direction
App: Application for an online or mobile technology
EPA: Enduring power of attorney
EPG: Enduring power of guardianship
MOOC: Massive Open Online Course
MPA: Medical power of attorney
RCT: Randomised controlled trial
SA: South Australia
SMS: Short message service
UID: Unique identifier number
