# Supplementary material for: Which online format is most effective for assisting Baby Boomers to complete advance directives? A randomised controlled trial of email prompting versus online education module
Source: BMC Palliat Care. 2017 Aug 29;16:43. doi: 10.1186/s12904-017-0225-9 (PMC5576351; doi:10.1186/s12904-017-0225-9)
Supplement: Supplementary file 1 — Diagram of 2 × 2 factorial design of RCT. (DOCX 12 kb) [file 12904_2017_225_MOESM1_ESM.docx]

|  | | Intervention A – AD Module | |
| --- | --- | --- | --- |
| Intervention B – email Prompt |  | No | Yes |
|  | No | No AD Module  No email Prompt  (Group A) | AD Module Only  (Group B) |
|  | Yes | email Prompt Only  (Group C) | email Prompt  AD Module  (Control Group D) |
| Fig. 1  Diagram of 2 x 2 factorial design of RCT | | | |
