# Supplementary material for: Which online format is most effective for assisting Baby Boomers to complete advance directives? A randomised controlled trial of email prompting versus online education module
Source: BMC Palliat Care. 2017 Aug 29;16:43. doi: 10.1186/s12904-017-0225-9 (PMC5576351; doi:10.1186/s12904-017-0225-9)
Supplement: Supplementary file 2 — Pre-survey for RCT. (DOCX 22 kb) [file 12904_2017_225_MOESM2_ESM.docx]

| **ADVANCE DIRECTIVES** |
| --- |
| **Welcome to the Boomer/Advance Directive Project**  In this survey you will be asked a series of questions which explore your current use of advance directives. An advance directive is a legally binding document that expresses a person’s instructions for future finances, healthcare and lifestyle in the event that mental capacity is lost. There are currently 4 different advance directive documents in South Australia. You will be asked questions about your knowledge and use of these documents. This survey will also ask questions about some of your personal details to establish baseline characteristics of participants involved in this study. |
| **Participant UID** |
| 1. **Have you completed any of the following documents for yourself?** *Please tick all answers that apply* 2. Enduring Power of Attorney (for finances) 3. Power of Attorney (for finances) 4. Enduring Power of Guardianship (for healthcare and lifestyle) 5. Will (for after your death) 6. Medical Power of Attorney (for medical treatment only) 7. Anticipatory Direction 8. Living Will 9. Advance Care Plan 10. Statement of Choices 11. Life Values Statement 12. Organ Donation Card 13. Ulysses Agreement or Psychiatric Advance Directive 14. Have not completed any of these types of instruments 15. None of the above 16. Prefer not to answer 17. Other – please describe |
| 1. **If you have completed any of the documents listed in Question 2, did you seek assistance from any of the following to complete the document** *Please tick all answers that apply* 2. Family member 3. Friend 4. Lawyer or Solicitor 5. Financial Planner 6. Justice of the Peace 7. Doctor or other Medical Specialist 8. Nurse 9. Allied Health Worker (such as physiotherapist, occupational therapist, podiatrist, speech pathologist or other) 10. Pharmacist 11. Personal Care Worker (or Assistant in Nursing) 12. Social Worker or Counsellor 13. Chaplain or Spiritual Guide 14. Complementary Therapist 15. Work Colleague 16. Website 17. Facebook or Social Network Friends 18. Did not seek assistance from anyone 19. None of the above 20. Prefer not to answer 21. Other – Please describe |
| 1. **When you heard about this project, did you discuss your thoughts on advance directives with any of the following?** *Please tick all answers that apply*   1 Family member  2 Friend  3 Lawyer or Solicitor  4 Financial Planner  5 Justice of the Peace  6 Doctor or other Medical Specialist  7 Nurse  8 Allied Health Worker (such as physiotherapist, occupational therapist, podiatrist, speech pathologist or other)  9 Pharmacist  10 Personal Care Worker (or Assistant in Nursing)  11 Social Worker or Counsellor  12 Chaplain or Spiritual Guide  13 Complementary Therapist  14 Work Colleague  15 Website  16 Facebook or Social Network Friends  17 Did not seek assistance from anyone  18 None of the above  19 Prefer not to answer  20 Other – Please describe |
| 1. **Thinking about your family and friends, have you helped anyone complete any of the following documents?** *Please tick all answers that apply*   1 Enduring Power of Attorney (for finances)  2 Power of Attorney (for finances)  3 Enduring Power of Guardianship (for healthcare and lifestyle)  4 Will (for after your death)  5 Medical Power of Attorney (for medical treatment only)  6 Anticipatory Direction  7 Living Will  8 Advance Care Plan  9 Statement of Choices  10 Life Values Statement  11 Organ Donation Card  12 Ulysses Agreement or Psychiatric Advance Directive  13 Have not helped anyone  14 None of the above  15 Prefer not to answer  16 Other – please |
| 1. **Have you ever acted as the Substitute Decision-Maker (SDM) for someone using any of the following documents?** 2. Yes – Enduring Power of Attorney 3. Yes – Power of Attorney 4. Yes – Enduring Power of Guardianship 5. Yes – Power of Attorney and Enduring Power of Guardianship 6. Yes – Enduring Power of Attorney and Enduring Power of Guardianship 7. Yes – Medical Power of Attorney 8. Yes – Enduring Power of Attorney and Medical Power of Attorney 9. Yes – Power of Attorney and Medical Power of Attorney 10. Yes – Enduring Power of Guardianship and Medical Power of Attorney 11. Yes – Power of Attorney, Enduring Power of Guardianship, Medical Power of Attorney 12. Yes – Enduring Power of Attorney, Medical Power of Attorney and Enduring Power of Guardianship 13. Guardianship Order 14. Yes – Ulysses Agreement or Psychiatric Advance Directive 15. Not sure 16. No 17. Prefer not to answer 18. Other – Please describe |
| **COMPUTER USE** |
| **This next section asks questions about your comfort with computers and use of the Internet.** |
| 1. **On a scale from 0% to 100%, how comfortable are you using a computer?** *Please tick the one box that best describes your comfort level with using a computer*   **0 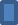 10 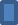 20 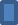 30 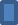 40 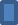 50 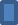 60 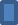 70 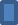 80 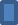 90 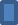 100** |
| 1. **On a weekly basis, how often do you use your computer?** *Please tick the one box that best describes your current weekly use of the computer at work, home or both*   **Once a week or less 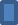 Two to three times a week 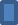 More than three times a week 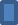** |
| 1. **On a scale from 0% to 100%, how comfortable are you using the Internet?** *Please tick the one box that best describes your comfort level with using the Internet*   **0 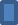 10 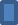 20 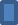 30 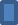 40 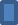 50 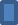 60 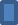 70 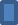 80 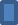 90 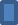 100** |
| 1. **On a weekly basis, how often do you use the Internet for things like email, banking, online shopping, googling for information or any other activities?** *Please tick the one box that best describes your current weekly use of the Internet either at work, home or both*   **Once a week or less 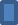 Two to three times a week 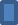 More than three times a week 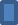** |
| 1. **Do you currently use any of the following social networks?** *Please tick all answers that apply*   1 Facebook (or similar)  2 Twitter (or similar)  3 YouTube  4 Chat Rooms  5 Blogs  6 I use Email for social networking  7 I don’t use online social networks  8 None of the above  9 Prefer not to answer  10 Other – Please describe |
| 1. **Which of the following devices do you use on a daily or weekly basis?** *Please tick all answers that apply*   1 Desktop Computer (PC or MAC)  2 Smartphone (iPhone or other)  3 Laptop or Notebook Computer  4 Tablet Device (iPad or other)  5 Kindle or other e-reader  6 Mobile Phone that is not a smartphone  7 None of the above  8 Prefer not to answer  9 Other – Please describe |
| 1. **Which of the following software applications would you use on a regular basis for home use?** *Please tick all answers that apply*   1 Word or similar word processing software  2 Excel or similar basic mathematical software  3 Publisher or other media software  4 Apps such as those found on iPhones, iPads, Android, etc.  5 Genealogy or Family History software  6 Online register for keeping documents  7 Education software for teaching or learning  8 Software for Professional Development  9 Skype  10 None of the above  11 Prefer not to answer  12 Other – Please describe |
| 1. **Which of the following would you find helpful if you wanted to learn more about advance directives?** *Please tick all answers that apply* 2. Information on the Internet 3. Online Advance directive forms 4. Online training on how to complete ADs 5. Online training on how and when to use an AD 6. Online register to put my ADs 7. Healthcare professionals online to answer questions about ADs 8. Telephone consultation 9. Prefer other ways to learn about ADs such as face-to-face 10. I am not interested in learning about ADs 11. None of the above 12. Prefer not to answer 13. Other – Please describe |
| **DEMOGRAPHIC INFORMATION** |
| **This next section requires personal detail information. This information is required for assessing your answers against those made by others who may share similar characteristics. Please answer as many as you can but if you prefer not to answer please click the “Prefer not to answer” box.** |
| 1. **Gender** Please tick only one box 2. Male 3. Female 4. Prefer not to answer |
| 1. **Year you were born** Please tick only one box 2. 1946 3. 1947 4. 1948 5. 1949 6. 1950 7. 1951 8. 1952 9. 1953 10. 1954 11. 1955 12. 1956 13. 1957 14. 1958 15. 1959 16. 1960 17. 1961 18. 1962 19. 1963 20. 1964 21. 1965 22. None of the above 23. Don’t know |
| 1. **Do you identify as an Aboriginal or Torres Strait Islander?** *Please tick only one box* 2. Yes 3. No 4. Prefer not to answer |
| 1. **What region of the world were you born in?** *Please tick only one box* 2. Australia and New Zealand 3. UK and Ireland 4. Europe 5. Asia 6. Africa 7. North America 8. South America 9. Oceania (for example Vanuatu, Fiji, Philippines or other) 10. Prefer not to answer 11. Other – Please describe |
| 1. **What is your marital status?** *Please tick only one box* 2. Married 3. De Facto 4. Separated 5. Divorced 6. Widowed 7. Single 8. Prefer not to answer |
| 1. **What is your current yearly household income** *Please tick only one box* 2. $180,001 or more 3. $160,001 – $180,000 4. $140,001 – $160,000 5. $120,001 – $140,000 6. $100,001 – $120,000 7. $80,001 – $100,000 8. $60,001 – $80,000 9. $40,001 – $60,000 10. $20,001 – $40,000 11. Under $20,000 12. Prefer not to answer |
| 1. **Which group best describes your current employment** *Please tick only one box* 2. Farming or Other Agricultural Employment 3. Art or Media 4. Business, Human Resource or Marketing 5. Design, Engineering, Science or Transport 6. Teacher (primary or secondary) 7. Health Professional (all types) 8. Information Technologist (ICT, Software or Hardware, or Analysis) 9. Legal, Social or Welfare 10. Technician (any kind) 11. Trades (any kind) 12. Apprentice (any kind) 13. Community and Personal Service 14. Clerical and Administrative 15. Sales (any kind) 16. Machine Operator or Driver (any kind) 17. Labourer (any kind) 18. Academic 19. Sportsperson 20. Student (no employment) 21. Housewife 22. Carer (Paid or Unpaid) 23. Retired 24. Currently not employed 25. None of the above 26. Prefer not to answer 27. Other – Please describe |
| 1. **In which part of South Australia do you currently live?** *Please tick only one box* 2. Metropolitan Adelaide (Adelaide and surrounding suburbs) 3. Rural or Regional (Adelaide Hills, Barossa, Eyre Peninsula and Western South Australia, Far North, Fleurieu Peninsula and Kangaroo Island, Limestone Coast, Murray Mallee, Yorke Peninsula and Mid North) 4. Prefer not to answer 5. Other – Please describe |
| 1. **Finally – can you please tell me how you learned about this study?** *Please tick all answers that apply*   1 Flier posted on a noticeboard  2 Electronic newsletter  3 Email  4 Paper newsletter  5 Word of mouth  6 Friend  7 Family member  8 Work or School Colleague  9 Health Omnibus Survey  10 Newspaper  11 None of the above  12 Prefer not to answer  13 Other – Please describe |
| Fig. 2  Pre-survey for RCT |
