# Supplementary figures and images for: Which online format is most effective for assisting Baby Boomers to complete advance directives? A randomised controlled trial of email prompting versus online education module
Source: BMC Palliat Care. 2017 Aug 29;16:43. doi: 10.1186/s12904-017-0225-9 (PMC5576351; doi:10.1186/s12904-017-0225-9)

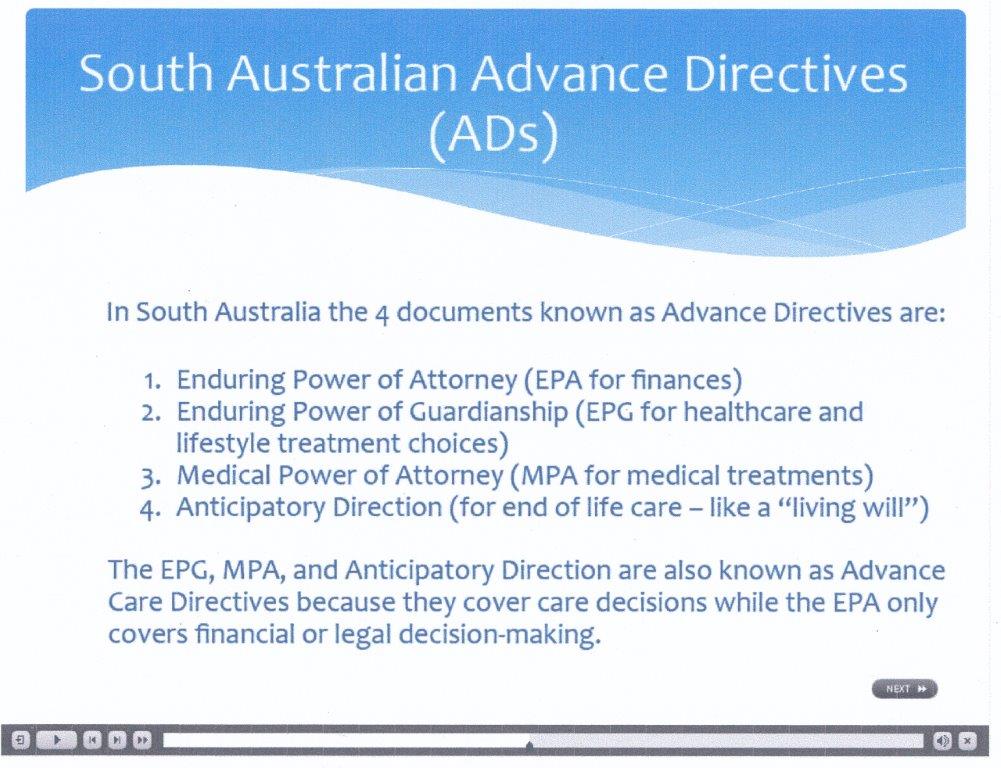


Fig. 3

Sample page of Education Module

Supplement: Supplementary file 3 — Sample page from online education module. (DOCX 119 kb) [file 12904_2017_225_MOESM3_ESM.docx]

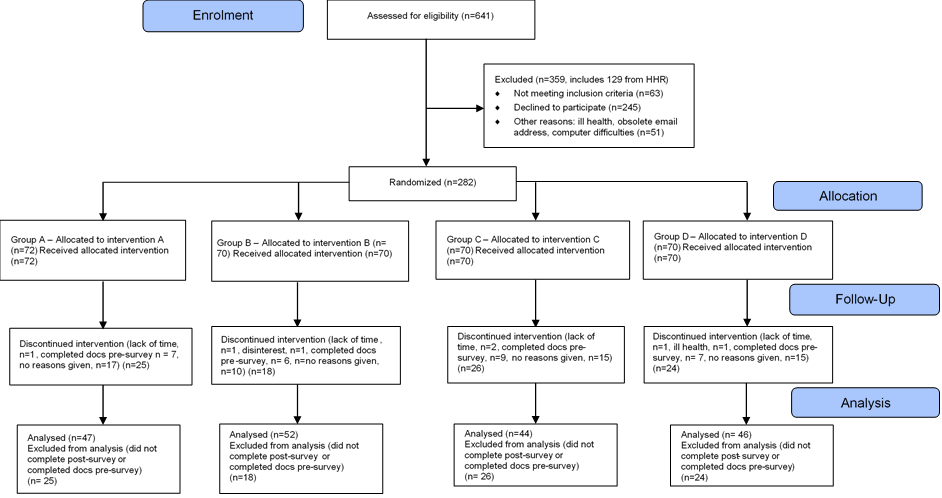


Fig. 6

Flow diagram of inclusions and exclusions based on CONSORT recommendations

Supplement: Supplementary file 6 — Flow diagram of inclusions and exclusions based on CONSORT recommendations. (DOCX 107 kb) [file 12904_2017_225_MOESM6_ESM.docx]
