# Supplementary material for: Which online format is most effective for assisting Baby Boomers to complete advance directives? A randomised controlled trial of email prompting versus online education module
Source: BMC Palliat Care. 2017 Aug 29;16:43. doi: 10.1186/s12904-017-0225-9 (PMC5576351; doi:10.1186/s12904-017-0225-9)
Supplement: Supplementary file 4 — Email Survey sent as Prompt for completing AD. (DOCX 15 kb) [file 12904_2017_225_MOESM4_ESM.docx]

| **ADVANCE DIRECTIVES** |
| --- |
| **This section will ask you for responses to questions about advance directives. An advance directive is a legally binding document that expresses a person’s wishes or directions in advance in the event that mental capacity is lost in the future. There are currently different documents in South Australia to cover specific areas of decision making** (Office of the Public Advocate of South Australia, 2011) |
| 1. **Have you completed any of the following forms for yourself since beginning this study?** *Circle all of the ones you have done* 2. Power of Attorney (for finances) 3. Power of Guardianship (for healthcare and welfare) 4. Will (for after you have died) 5. Medical Power of Attorney (for healthcare) 6. “Living Will” 7. Advance Care Plan 8. Statement of Choices 9. Good Palliative Care Plan 10. Life Values Statement 11. Organ donation card 12. Other – please describe 13. None of the above 14. Prefer not to answer |
| 1. **If you have completed any of the documents listed in Item 1, did you seek assistance from any of the following (**circle all of the ones that assisted you): 2. Family member 3. Friend 4. Lawyer or Solicitor 5. Financial Planner 6. Doctor or any other Healthcare Professional (nurse, social worker, physiotherapist, occupational therapist) 7. Website with information on advance directives 8. Other (please specify: ) 9. None of the above 10. Prefer not to answer |
| 1. **Since participating in this study, have you discussed advance directives or the need to do them with (**please circle all that apply) 2. Family member 3. Friend 4. GP or other medical practitioner 5. Nurse or other healthcare professional 6. Work colleague 7. Facebook or other Social Media Friends 8. Spiritual Counsellor (for example, Minister, Chaplain, Elder) 9. Social Worker 10. Prefer not to answer |
| 1. **Thinking about your family and friends, have you helped someone learn about or complete any of the following forms since the commencement of this study?** *Circle all of the ones you have helped someone else with* 2. Power of Attorney (for finances) 3. Power of Guardianship (for healthcare and welfare) 4. Will (for after you have died) 5. Medical Power of Attorney (for healthcare) 6. “Living Will” 7. Advance Care Plan 8. Statement of Choices 9. Good Palliative Care Plan 10. Life Values Statement 11. Organ donation card 12. Other – please describe 13. None of the above 14. Prefer not to answer |
| 1. **Since the commencement of this study, have you acted as the power of attorney or guardianship for someone else?** 2. Yes 3. No 4. Prefer not to answer |
| Fig. 4  Email Survey sent as Prompt for completing AD |
