# Supplementary material for: Which online format is most effective for assisting Baby Boomers to complete advance directives? A randomised controlled trial of email prompting versus online education module
Source: BMC Palliat Care. 2017 Aug 29;16:43. doi: 10.1186/s12904-017-0225-9 (PMC5576351; doi:10.1186/s12904-017-0225-9)
Supplement: Supplementary file 5 — Post Survey (DOCX 21 kb) [file 12904_2017_225_MOESM5_ESM.docx]

| **ADVANCE DIRECTIVES** |
| --- |
| **Welcome to the Final Survey in the Boomer/Advance Directive Project**  So we can identify any differences that occurred from the beginning of the study to its conclusion, in this final survey you will be asked a series of questions which again explore your use of advance directives. You will also be asked questions about your use of the online environment in assisting you with gaining more information about these documents and/or completing them. |
| **Participant UID** |
| 1. **Have you completed any of the following documents for yourself?** *Please tick all answers that apply* 2. Enduring Power of Attorney (for finances) 3. Power of Attorney (for finances) 4. Enduring Power of Guardianship (for healthcare and lifestyle) 5. Will (for after your death) 6. Medical Power of Attorney (for medical treatment only) 7. Anticipatory Direction 8. Living Will 9. Advance Care Plan 10. Statement of Choices 11. Life Values Statement 12. Organ Donation Card 13. Ulysses Agreement or Psychiatric Advance Directive 14. Have not completed any of these types of instruments 15. None of the above 16. Prefer not to answer 17. Other – please describe |
| 1. **If you have not completed any of the documents described in Question 1** (*researcher note: should have been Question 2)*, **could you please explain why?** *The choices below are examples of what previous respondents have identified – if none of these apply to you, please choose “Other-please describe” and explain what stopped you from completing them. Please tick all answers that apply.*   1 Too Busy  2 Not the right time  3 Couldn’t get the documents  4 Couldn’t choose a substitute decision-maker  5 Didn’t have anyone to discuss with  6 Couldn’t find a witness  7 Couldn’t understand the forms  8 Needed more information  9 Don’t feel the need to complete them  10 Prefer the doctor to make decisions  11 Prefer the family to make decisions  12 Against my religious beliefs  13 Against my cultural beliefs  14 Prefer not to answer  15 Other – Please describe |
| 1. **If you have completed any of the documents listed in Question 2, did you seek assistance from any of the following to complete the document** *Please tick all answers that apply* 2. Family member 3. Friend 4. Lawyer or Solicitor 5. Financial Planner 6. Justice of the Peace 7. Doctor or other Medical Specialist 8. Nurse 9. Allied Health Worker (such as physiotherapist, occupational therapist, podiatrist, speech pathologist or other) 10. Pharmacist 11. Personal Care Worker (or Assistant in Nursing) 12. Social Worker or Counsellor 13. Chaplain or Spiritual Guide 14. Complementary Therapist 15. Work Colleague 16. Website 17. Facebook or Social Network Friends 18. Did not seek assistance from anyone 19. None of the above 20. Prefer not to answer 21. Other – Please describe |
| 1. **Since participating in this project, have you discussed your thoughts on advance directives with any of the following?** *Please tick all answers that apply*   1 Family member  2 Friend  3 Lawyer or Solicitor  4 Financial Planner  5 Justice of the Peace  6 Doctor or other Medical Specialist  7 Nurse  8 Allied Health Worker (such as physiotherapist, occupational therapist, podiatrist, speech pathologist or other)  9 Pharmacist  10 Personal Care Worker (or Assistant in Nursing)  11 Social Worker or Counsellor  12 Chaplain or Spiritual Guide  13 Complementary Therapist  14 Work Colleague  15 Facebook or Social Network Friends  16 Did not discuss with anyone  17 None of the above  18 Prefer not to answer  19 Other – Please describe |
| 1. **Thinking about your family and friends, have you helped anyone to complete any of the following documents since your participation in this study?** *Please tick all answers that apply*   1 Enduring Power of Attorney (for finances)  2 Power of Attorney (for finances)  3 Enduring Power of Guardianship (for healthcare and lifestyle)  4 Will (for after your death)  5 Medical Power of Attorney (for medical treatment only)  6 Anticipatory Direction  7 Living Will  8 Advance Care Plan  9 Statement of Choices  10 Life Values Statement  11 Organ Donation Card  12 Ulysses Agreement or Psychiatric Advance Directive  13 Have not helped anyone  14 None of the above  15 Prefer not to answer  16 Other – please |
| 1. **Have you ever acted as the Substitute Decision-Maker (SDM) for someone using any of the following documents?** *Please tick all answers that apply* 2. Yes – Enduring Power of Attorney 3. Yes – Power of Attorney 4. Yes – Enduring Power of Guardianship 5. Yes – Power of Attorney and Enduring Power of Guardianship 6. Yes – Enduring Power of Attorney and Enduring Power of Guardianship 7. Yes – Medical Power of Attorney 8. Yes – Enduring Power of Attorney and Medical Power of Attorney 9. Yes – Power of Attorney and Medical Power of Attorney 10. Yes – Enduring Power of Guardianship and Medical Power of Attorney 11. Yes – Power of Attorney, Enduring Power of Guardianship, Medical Power of Attorney 12. Yes – Enduring Power of Attorney, Medical Power of Attorney and Enduring Power of Guardianship 13. Guardianship Order 14. Yes – Ulysses Agreement or Psychiatric Advance Directive 15. Not sure 16. No 17. Prefer not to answer 18. Other – Please describe |
| 1. **Would you act as a substitute decision-maker if someone asked you to?**   1 Yes  2 No  3 Maybe  4 Depends on who asked  5 Prefer not to answer |
| 1. **Since participating in this research study, which of the following did you find particularly helpful when you wanted to learn more about advance directives?**   1 Information on the Internet  2 Online advance directive forms  3 Online training on how to complete ADs  4 Online training on how and when to use an AD  5 Online register to put my ADs  6 Apps  7 Healthcare professionals online to answer questions about ADs  8 Telephone consultation with knowledgeable professional  9 URL links to information from websites  10 Videos about ADs, e.g. YouTube  11 The surveys in this research study  12 Emails sent during the study prompted me to learn more about completing ADs  13 Prefer other ways to learn about ADs such as face-to-face  14 Discussions with family or friends  15 I am not interested in learning about ADs  16 None of the above  17 Prefer not to answer  18 Other – Please describe |
| 1. **Since participating in this research study, which of the following did you find particularly helpful when you wanted to complete advance directives?**   1 Information on the Internet  2 Online advance directive forms  3 Online training on how to complete ADs  4 Online training on how and when to use an AD  5 Online register to put my ADs  6 Healthcare professionals online to answer questions about ADs  7 Face-to-face discussion with knowledgeable professionals  8 Discussions with family or friends  9 Telephone consultation with knowledgeable professional  10 URL links to information from websites  11 Videos about ADs, e.g. YouTube  12 Emails sent during the study prompted me to learn more about completing ADs  13 I do not intend to complete any ADs  14 None of the above  15 Prefer not to answer  16 Other – Please describe |
| 1. **Have any of the following devices assisted you to learn more about advance directives or to complete the documents?** *Please tick all answers that apply*   1 Desktop Computer (PC or MAC)  2 Smartphone (iPhone or other)  3 Laptop or Notebook Computer  4 Tablet Device (iPad or other)  5 Kindle or other e-reader  6 Mobile Phone that is not a smartphone  7 Television  8 None of the above  9 Prefer not to answer  10 Other – Please describe |
| 1. **Have any of the following software applications helped you to learn about advance directives?** *Please tick all answers that apply*   1 Word or similar word processing software  2 Excel or similar basic mathematical software  3 Publisher or other media software  4 Apps such as those found on iPhones, iPads, Android, etc.  5 Genealogy or Family History software  6 Online register for keeping documents  7 Education software for teaching or learning  8 Software for Professional Development  9 Skype  10 CareSearch Website  11 Other Website  12 None of the above  13 Prefer not to answer  14 Other – Please describe |
| 1. **If you currently use social networks and could get reminders to complete advance directives, which of the following networks would you prefer reminders be sent to?**   1 Facebook (or similar)  2 Twitter (or similar)  3 SMS or text messaging  4 Email  5 Television Advertisement  6 Advertisement in cinema  7 Advertisement on websites  8 Don’t want to be reminded  9 None of the above  10 Prefer not to answer  11 Other – Please describe |
| 1. **If you wanted to be reminded to complete advance directives, when would it be the best time for a reminder to be sent to you?** *Please tick only one box* 2. Your birthday 3. When your car registration is due 4. During a visit to your GP or local clinic 5. During a visit to the hospital 6. When getting or renewing a passport 7. When travelling overseas 8. When completing a Will 9. When visiting a financial planner or lawyer/solicitor 10. When completing an organ donation card 11. When completing an advance directive for someone else 12. On your 40^th^ birthday 13. On your 50^th^ birthday 14. On your 60^th^ birthday 15. On your 70^th^ birthday or older 16. Don’t want reminders 17. Prefer not to answer 18. Other – Please describe |
| **Ok – Final Section – general questions about the survey structure and design** |
| 1. **Did you find the online surveys (like the Pre-survey and this survey) easy to use?** *Please tick only one box* 2. Yes 3. No 4. Sometimes 5. Not sure 6. Prefer not to answer |
| 1. **If you did NOT find the online surveys (like the Pre-survey and this survey) easy to use, could you please describe in detail the difficulties you had with using these online surveys. The more detail you provide, the better future surveys can be made to meet the needs of other consumers such as yourself.** *Please describe as clearly and in as much detail as possible* |
| 1. **How do you think online information about advance directives could be improved?** *Please tick all answers that apply* 2. Make terms clearer 3. Use language I understand 4. Give more examples 5. Provide better reasons for completing them 6. Have direct access to the forms 7. Provide information on how to have a conversation about advance directives 8. Provide guidelines for choosing a substitute decision-maker 9. Tell me who to go to when I can’t find a substitute decision-maker 10. Tell me more specifically when I should do advance directives 11. More graphs, figures or illustrations to describe information 12. Show successful use of advance directives in different situations 13. Testimonials from healthcare professionals, families, carers and people who have created or used advance directives 14. Show famous people describing their experience with advance directives 15. Prefer not to answer 16. Other – Please describe |
| 1. **Was there anything that you experienced during the course of this study that heightened your awareness of advance directives? If so, please explain what this was. Please also include the names of any websites you visited to learn more about advance directives.** |
| 1. **Would you agree that you now know more about advance directives?** *Please tick only one box*   1 Yes  2 No  3 Maybe  4 Not sure  5 Don’t Know  6 Prefer not to answer |
| 1. **How likely are you to complete any advance directive document in the next three (3) months?** *Please tick only one box*   Unlikely 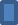 Maybe 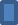 Very likely 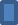 Am in the process of completing 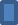 Have already completed documents 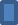  Am not interested in completing advance directives 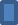 |
| 1. **Are there any other comments you would like to make about this study, the surveys or advance directives in general?** *Please describe as clearly and in as much detail as possible* |
| Fig. 5  Post Survey |
