# Supplementary material for: Which online format is most effective for assisting Baby Boomers to complete advance directives? A randomised controlled trial of email prompting versus online education module
Source: BMC Palliat Care. 2017 Aug 29;16:43. doi: 10.1186/s12904-017-0225-9 (PMC5576351; doi:10.1186/s12904-017-0225-9)
Supplement: Supplementary file 7 — Demographics of 189 Participants in RCT. (DOCX 20 kb) [file 12904_2017_225_MOESM7_ESM.docx]

Appendix 7

Table 1: Demographics of 189 Participants in RCT

|  | Overall  N=189 | % (100) | Group A  N=47  n (%) | Group B  N=52  n (%) | Group C  N=44  n (%) | Group D  N=46  n (%) | P value | Combined Group Prompt (C+D=90)  n % | Combined Group Non-Prompt (A+B=99)  n (%) | P  value | Combined Group AD Module (B+D=98)  n (%) | Combined Non-AD Module (A+C=91)  n % | P value |
| --- | --- | --- | --- | --- | --- | --- | --- | --- | --- | --- | --- | --- | --- |
| **Birth Decade** |  |  |  |  |  |  |  |  |  |  |  |  |  |
| 1946–1955 | 103 | 54% | 28 (60) | 28 (54) | 25 (57) | 22 (48) |  | 47 (52) | 56 (57) |  | 50 (51) | 53 (58) |  |
| 1956–1965 | 86 | 46% | 19 (40) | 24 (46) | 19 (43) | 24 (52) | 0.70 | 43 (48) | 43 (43) | 0.55 | 48 (49) | 38 (42) | 0.32 |
| **Gender** |  |  |  |  |  |  |  |  |  |  |  |  |  |
| Male | 48 | 25% | 10 (21) | 14 (27) | 13 (30) | 11 (24) |  | 24 (27) | 24 (24) |  | 25 (26) | 23 (25) |  |
| Female | 141 | 75% | 37 (79) | 38 (73) | 31 (71) | 35 (76) | 0.82 | 66 (73) | 75 (76) | 0.70 | 73 (75) | 68 (75) | 0.97 |
| **Location** |  |  |  |  |  |  |  |  |  |  |  |  |  |
| Metropolitan | 143 | 76% | 38 (81) | 36 (69) | 34 (77) | 35 (76) |  | 69 (77) | 74 (75) |  | 71 (72) | 72 (79) |  |
| Rural | 46 | 24% | 9 (19) | 16 (31) | 10 (23) | 11 (24) | 0.59 | 21 (23) | 25 (25) | 0.76 | 27 (28) | 19 (21) | 0.29 |
| **Country of Birth** |  |  |  |  |  |  |  |  |  |  |  |  |  |
| Australia and New Zealand | 136 | 72% | 30 (64) | 39 (75) | 35 (80) | 32 (70) |  | 67 (74) | 69 (70) |  | 71 (72) | 65 (71) |  |
| Other Country | 53 | 28% | 17 (36) | 13 (25) | 9 (21) | 14 (30) | 0.37 | 23 (26) | 30 (30) | 0.47 | 27 (28) | 26 (29) | 0.88 |
| **Marital Status** |  |  |  |  |  |  |  |  |  |  |  |  |  |
| Married/De Facto | 114 | 61% | 24 (51) | 37 (71) | 27 (61) | 26 (57) |  | 53 (59) | 61 (62) |  | 63 (64) | 51 (56) |  |
| Sep/Divorced/Widow/  Single | 73 | 38% | 23 (49) | 15 (29) | 16 (36) | 19 (41) | 0.35 | 35 (39) | 38 (38) | 0.32 | 34 (35) | 39 (43) | 0.51 |
| **Occupation** |  |  |  |  |  |  |  |  |  |  |  |  |  |
| Professionals | 74 | 39% | 23 (49) | 19 (37) | 13 (30) | 19 (41) |  | 32 (36) | 42 (42) |  | 38 (39) | 36 (40) |  |
| Clerical/Sales | 40 | 22% | 9 (19) | 12 (23) | 12 (27) | 7 (15) |  | 19 (21) | 21 (21) |  | 19 (19) | 21 (23) |  |
| Blue Collar | 6 | 2% | 0 (0) | 2 (4) | 0 (0) | 4 (9) |  | 4 (4) | 2 (2) |  | 6 (6) | 0 (0) |  |
| Never Worked/Student/  Home Duties/Retired | 68 | 36% | 15 (32) | 18 (35) | 19 (43) | 16 (35) | 0.24 | 35 (39) | 33 (33) | 0.60 | 34 (35) | 34 (37) | 0.14 |
| **Annual Income*** |  |  |  |  |  |  |  |  |  |  |  |  |  |
| $80,001+ | 73 | 39% | 11 (23) | 11 (21) | 13 (30) | 9 (20) |  | 31 (34) | 42 (42) |  | 35 (39) | 38 (42) |  |
| $40,001–$80,000 | 48 | 25% | 13 (28) | 11 (21) | 6 (14) | 18 (39) |  | 24 (26) | 24 (24) |  | 29 (32) | 19 (21) |  |
| $0–$40,000 | 44 | 23% | 19 (40) | 23 (44) | 19 (43) | 12 (26) | 0.28 | 22 (24) | 22 (22) | 0.71 | 20 (22) | 24 (26) | 0.40 |

*Not all participants answered this question so percentage does not add up to 100%
