# Supplementary material for: Which online format is most effective for assisting Baby Boomers to complete advance directives? A randomised controlled trial of email prompting versus online education module
Source: BMC Palliat Care. 2017 Aug 29;16:43. doi: 10.1186/s12904-017-0225-9 (PMC5576351; doi:10.1186/s12904-017-0225-9)
Supplement: Supplementary file 8 — Primary outcomes of individuals and individual documents completed per group from Pre-survey to Post-survey. (DOCX 14 kb) [file 12904_2017_225_MOESM8_ESM.docx]

Appendix 8

Table 2: Primary outcomes of individuals and individual documents completed per group from Pre-survey to Post-survey

| Individuals who completed one or more documents  n (%)  /Types of Documents completed | All Groups (N=189) | | Individuals Completing Documents Prompt Group (C+D, N=90) n=6 (%) | Document completions Prompt Group | | Individuals Completing Documents Non-Prompt Group (A+B, N=99)  n=7 (%) | Document completions Non-Prompt Group | | Individuals Completing Documents AD Module Group (B+D, N=98)  n=9 (%) | Document completions  AD Module Group | | Individuals Completing Documents Non-AD Module Group (A+C, N=91)  n=4 (%) | Document completions  Non-AD Module Group | |
| --- | --- | --- | --- | --- | --- | --- | --- | --- | --- | --- | --- | --- | --- | --- |
| 13 (7%) | Pre | Post | 6 (6%) | Pre | Post | 7 (7%) | Pre | Post | 9 (9%) | Pre | Post | 4 (4%) | Pre | Post |
| EPA | 0 | 7 |  | 0 | 3 |  | 0 | 4 |  | 0 | 6 |  | 0 | 1 |
| EPG | 0 | 6 |  | 0 | 2 |  | 0 | 4 |  | 0 | 4 |  | 0 | 2 |
| MPA | 0 | 5 |  | 0 | 1 |  | 0 | 4 |  | 0 | 2 |  | 0 | 3 |
| Ant Dir | 0 | 2 |  | 0 | 0 |  | 0 | 2 |  | 0 | 1 |  | 0 | 1 |
| LW | 0 | 2 |  | 0 | 1 |  | 0 | 1 |  | 0 | 1 |  | 0 | 1 |
| Total Docs | 0 | 22 |  | 0 | 7 |  | 0 | 15 |  | 0 | 14 |  | 0 | 8 |
