# Supplementary material for: Which online format is most effective for assisting Baby Boomers to complete advance directives? A randomised controlled trial of email prompting versus online education module
Source: BMC Palliat Care. 2017 Aug 29;16:43. doi: 10.1186/s12904-017-0225-9 (PMC5576351; doi:10.1186/s12904-017-0225-9)
Supplement: Supplementary file 9 — Binary logistic regression analysis comparing Prompt/Non-Prompt v AD module/non-AD module groups AD completions (N = 189). (DOCX 13 kb) [file 12904_2017_225_MOESM9_ESM.docx]

Appendix 9

Table 4 Binary logistic regression analysis comparing Prompt/Non-Prompt v AD module /non-AD module groups AD completions ^§^ (N=189)

| Post-survey Q2A: Completion rates of any of the 4 legal ADs (EPA, EPG, MPA, Anticipatory Direction and Living Will same document but treated separately) | | | | | | | |
| --- | --- | --- | --- | --- | --- | --- | --- |
|  | | Prompt groups (Groups C+D) versus Non-Prompt groups (Groups A+B) | | | AD Module groups (B+D) versus Non-AD module groups (A+C) | | |
| Document Name | Number of individual documents completed  N^#^*=22 | Odds Ratio | 95% CI | P Value | Odds Ratio | 95% CI | P Value |
| EPA | 7 | .8 | [.18, 3.76] | .82 | 5.9 | [0.69, 49.63] | .11 |
| EPG | 6 | .5 | [0.10, 3.02] | .49 | 1.9 | [0.33, 10.55] | .47 |
| MPA | 5 | .3 | [0.03, 2.42] | .24 | .6 | [0.10, 3.70] | .58 |
| Ant Dir | 2 | 0 | 0 | 1 | .9 | [0.06, 14.84] | .94 |
| LW | 2 | 1.1 | [0.68, -17.85] | .95 | .9 | [0.57, -15.08] | .96 |

*N=rounded to whole number

#Number who responded

§ At pre-survey, none of these documents were allowed to be completed
