# Supplementary material for: Which online format is most effective for assisting Baby Boomers to complete advance directives? A randomised controlled trial of email prompting versus online education module
Source: BMC Palliat Care. 2017 Aug 29;16:43. doi: 10.1186/s12904-017-0225-9 (PMC5576351; doi:10.1186/s12904-017-0225-9)
Supplement: Supplementary file 11 [file 12904_2017_225_MOESM12_ESM.docx]

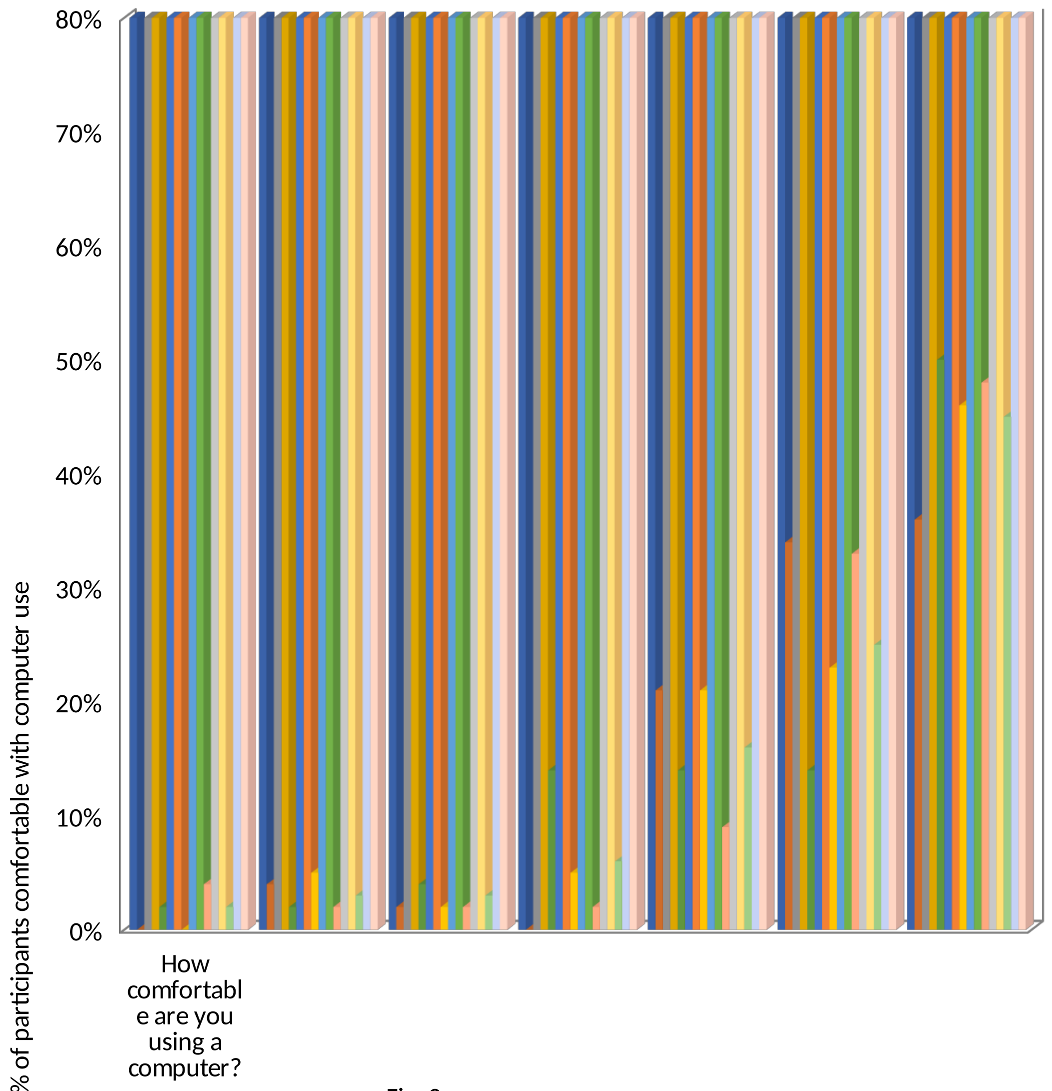

Fig. 9
Level of comfort individual has with using computer on a scale of 0-100%
First column is Group A, Control (n=46); second column Group B, AD Module (n=52); third column Group C, Prompt (n=44) and fourth column Group D, AD+prompt, n=46. Final column is

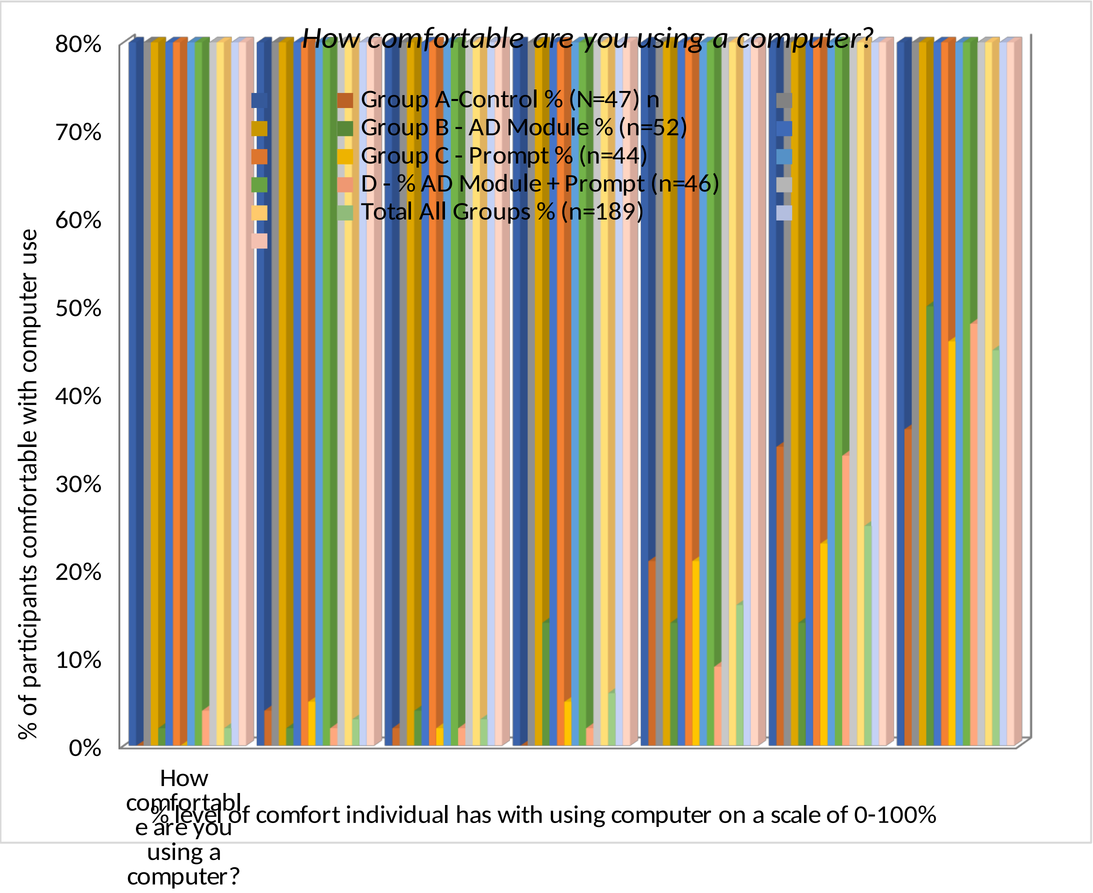
How comfortable are you using a computer?
Group A-Control % (N=47) n
Group B - AD Module % (n=52)
Group C - Prompt % (n=44)
D - % AD Module + Prompt (n=46)
Total All Groups % (n=189)
80%
70%
60%
50%
40%
30%
20%
10%
0%
% of participants comfortable with computer use
How comfortable are you using a computer?
% level of comfort individual has with using computer on a scale of 0-100%
