# Supplementary material for: Which online format is most effective for assisting Baby Boomers to complete advance directives? A randomised controlled trial of email prompting versus online education module
Source: BMC Palliat Care. 2017 Aug 29;16:43. doi: 10.1186/s12904-017-0225-9 (PMC5576351; doi:10.1186/s12904-017-0225-9)
Supplement: Supplementary file 13 [file 12904_2017_225_MOESM14_ESM.docx]

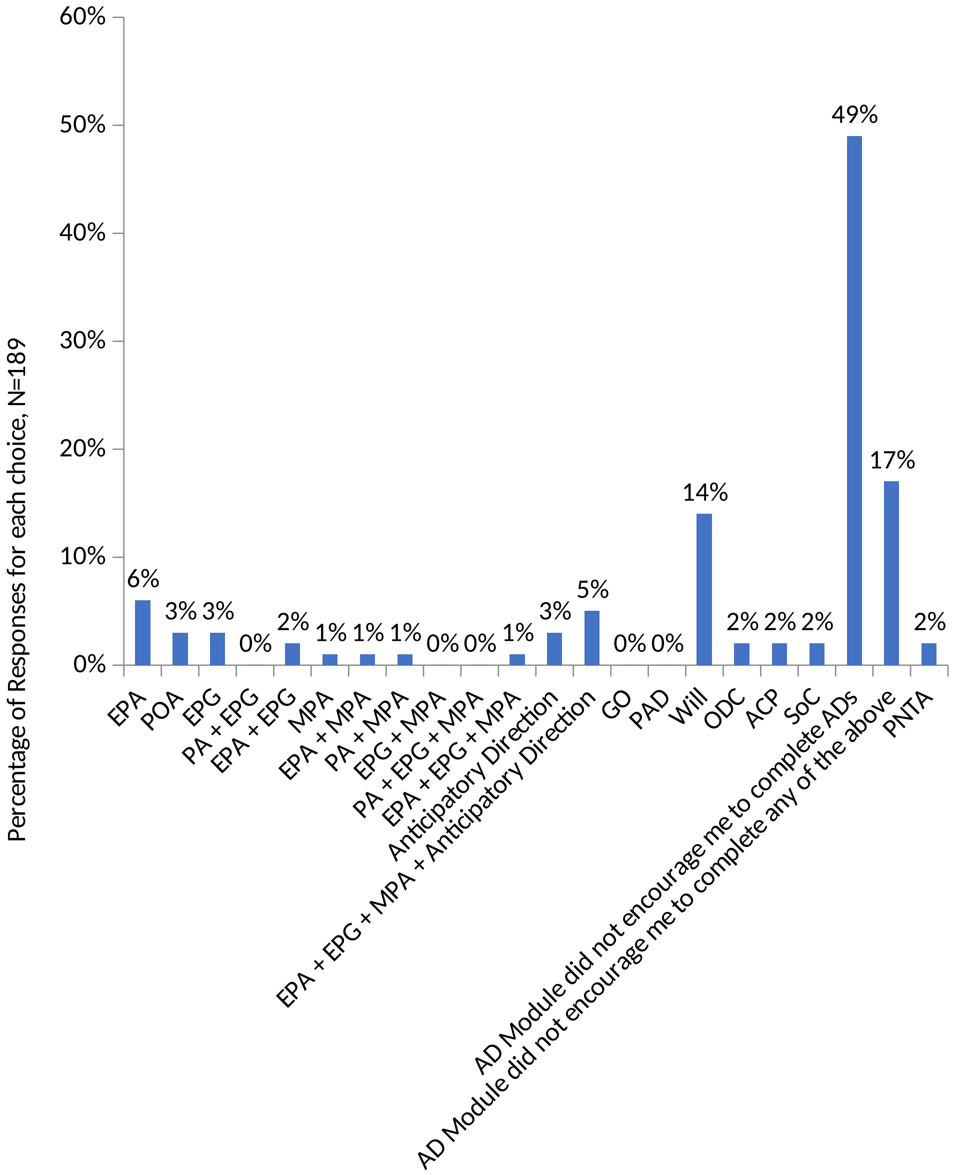

Fig. 11
Document Completions influenced by AD Module (PNTA=Prefer Not to Answer)
